# Unravelling the Genetic Architecture of Serum Biochemical Indicators in Sheep

**DOI:** 10.3390/genes15080990

**Published:** 2024-07-27

**Authors:** Mehmet Kizilaslan, Yunus Arzik, Sedat Behrem, Esra Yavuz, Stephen N. White, Mehmet Ulas Cinar

**Affiliations:** 1 Faculty of Agriculture, Department of Animal Science, Erciyes University, Kayseri 38039, Türkiye; mehmet.kizilaslan@tarimorman.gov.tr (M.K.); yunus.arzik@tarimorman.gov.tr (Y.A.); 2 International Center for Livestock Research and Training, Ministry of Agriculture and Forestry, Ankara 06852, Türkiye; 3 Department of Veterinary Sciences, Aksaray University, Aksaray 68100, Türkiye; 4 Department of Veterinary Microbiology & Pathology, College of Veterinary Medicine, Washington State University, Pullman, WA 99164, USA; stephen_white@wsu.edu

**Keywords:** serum, biochemical indicators, sheep, GWAS, QTL, genetic correlation, heritability

## Abstract

Serum biochemical indicators serve as vital proxies that reflect the physiological state and functions of different organs. The genetic parameters and molecular mechanisms underlying serum biochemical indicators of sheep (*Ovis aries*) have not been well understood. Therefore, the aim of the present study was to identify the genetic architecture and genomic loci underlying ten serum biochemical indicators in sheep, including alanine transaminase, aspartate transferase, lactate dehydrogenase, cholesterol, glucose, phosphorus, calcium, creatinine, urea and total protein levels. We implemented genetic parameter estimations and GWASs for each trait in 422 Akkaraman lambs. Overall, low to moderate heritability estimates were found in the range of 0.14–0.55. Additionally, low to high genetic correlations were observed among traits. In total, 23 SNP loci were associated with serum biochemical indicators leading to 19 genes. These were *SPTA1*, *MGST2*, *CACUL1*, *IGFBP7*, *PARD3*, *PHB1*, *SLC15A5*, *TRIM35*, *RGS6*, *NUP93*, *CNTNAP2*, *SLC7A11*, *B3GALT5*, *DPP10*, *HST2ST1*, *NRP1*, *LRP1B*, *MAP3K9* and *ENSOARG00020040484.1*, as well as *LOC101103187*, *LOC101117162*, *LOC105611309* and *LOC101118029.* To our knowledge, these data provide the first associations between *SPTA1* and serum cholesterol and between *ENSOARG00020040484.1* and serum glucose. The current findings provide a comprehensive inventory of the relationships between serum biochemical parameters, genetic variants and disease-relevant characteristics. This information may facilitate the identification of therapeutic targets and fluid biomarkers and establish a strong framework for comprehending the pathobiology of complex diseases as well as providing targets for sheep genetic improvement programs.

## 1. Introduction

Animals’ blood components reflect their immune systems and the metabolism of nutrients. Serum is the fluid and solute fraction of blood that lacks erythrocytes, platelets, leukocytes and clotting factors [1]. Serum contains a wide range of nutrients, including proteins and electrolytes, as well as antigens, antibodies, hormones and exogenous elements not needed for clotting. Serum is also required for the body’s delivery of nutrients, preservation of the homeostasis of the intracellular environment, and electrolyte and acid-base balance [2,3].

Serum biochemical indicators serve as vital proxies that reflect the physiological state and functions of different organs. As molecular phenotypic biomarkers, they are commonly employed as general indicators to assess an organism’s immunological status and overall health condition [4,5,6]. Many of these parameters appear to have moderate to high heritability in various species, including humans, pigs and horses [7,8,9]. Correspondingly, these traits are expected to be under tighter genetic control compared to associated diseases and complex traits, since they are directly linked to biochemical pathways, which might provide valuable information about the underlying biological control [7,10,11]. Therefore, identifying the genetic architecture responsible for their variability may contribute to a better understanding of the biological processes involved in various diseases and complex traits that are linked to these molecular phenotypes.

Animal welfare and health status are of paramount importance for all livestock enterprises, since any deviation from good health might have adverse effects on the profitability, productivity and sustainability of production systems [12]. Mounting evidence suggest that serum biochemical parameters have a wide range of associations with disease resistance, resilience, immune functions, productivity and feed efficiency in various livestock species [7,13,14,15,16,17]. Ruminant production in particular holds significant relevance in addressing two fundamental global challenges: (1) enhancing food security and nutrition for an expanding global population and (2) addressing the imperative of climate change mitigation [18,19]. However, conventional breeding strategies fall short of meeting expectations, especially for those traits that are difficult and costly to measure directly, including traits that are expressed later in life, such as disease resistance, immunity and longevity [5,20]. Nonetheless, the economic benefits of prioritizing disease resistance and robustness through genome-based selection are suggested to surpass the potential drawbacks of slower genetic progress in other traits in livestock [21]. Strong connections between serum biochemical markers and key livestock traits could enable their use as indicators for indirect selection across multiple traits, potentially overcoming drawbacks of traditional breeding methods.

Due to the rapid emergence of high-throughput sequencing and genotyping technologies, GWAS has become a widely used statistical approach to discover QTLs related to complex traits in various species, including humans, pigs, cattle, goats and sheep [4,7,22,23,24,25]. Genome-based selection methods are suggested to speed up genetic progress in selection schemes by reducing generation intervals and increasing the accuracy and intensity of selection in livestock production systems [26,27,28,29,30]. Sheep play a significant role in ensuring food security and sustainable production among livestock species thanks to their resilient adaptability and robust characteristics [19]. Furthermore, various studies suggest sheep as a more suitable model organism than rodents for investigating and establishing treatments for several human clinical conditions [31,32]. Therefore, dissection of the genetic basis underlying serum biochemical traits observed in sheep is a potential approach to designing a comprehensive marker-assisted selection program to prioritize sustainability, enhance resilience and support animal model development. To date, various genomic loci have been associated with serum biochemical parameters in humans as well as in livestock, such as pigs, cattle and ducks [4,5,7,10,33,34]. However, only one study has been identified that specifically investigated the genomic heritabilities and QTLs associated exclusively with serum protein levels in sheep [12].

Akkaraman sheep are an adaptive fat-tailed breed that represent an extensive share of Turkey’s sheep population and are spread throughout diverse terrains, from harsh, semi-arid regions to mild climates with comparatively moderate productivity characteristics [24]. Recently, the genome of the breed has been characterized against various world-wide sheep breeds to understand genomic relationships [35]. The large spread of the populations, the close genomic relationships with various sheep breeds, and its hardy and robust nature indicate the potential of the breed’s physiology for sustainable production under increased temperatures and extreme environmental conditions due to global warming. Therefore, the aim of the present study was to identify the genetic architecture and genomic loci underlying measurements of certain serum biochemical indicators in Akkaraman sheep, including alanine transaminase, aspartate transferase, lactate dehydrogenase, cholesterol, glucose, phosphorus, calcium, creatinine, urea and total protein levels. Our findings contribute insights into the genomic basis of complex serum biochemical traits that are of clinical and physiological importance.

## 2. Materials and Methods

The authors followed the ARRIVE guidelines and EU regulations on animal research throughout the research process [36]. Approval of the Local Ethics Committee for Experimental Animals of the Ministry of Agriculture and Forestry in Turkey, with the file number 20 November 2020/183, was obtained for use of the studied population.

### 2.1. Animal Population and Blood Serum Indicators

The experimental population consisted of 422 Akkaraman lambs (252 females and 170 males) of the study population described in [24]. Briefly, the animals were sourced from three commercial farms located in the district of Ankara, Turkey (39°41′ N, 33°01′ E). The region is characterized by harsh and cold winters and dry and parching heat during the summer, as well as meagre, poor-quality grassland. Phenotypic selection was applied across generations, where growth rate was considered for mating designs. The animals were registered with the National Small Ruminant Breeding Program. They were born between January and February 2021 and weaned between April and May 2021. Once weaned, 101 of the animals were maintained in a feedlot until six months of age, while the remaining 321 were fed by pasture grazing.

While collecting blood samples for the genotyping stage in EDTA-coated vacutainers at six months of age, a separate set of samples were collected in yellow biochemical analysis tubes with a gel and clot activator, reaching a total of 8–10 mL of blood sampled per animal. The yellow biochemical analysis tubes were immediately centrifuged at 4100 rpm for 5–6 min, and the serum was separated from the clot and stored in a −80 °C freezer until use. Serum samples were later sent to a private biochemistry lab for the measurement of alanine transaminase (ALT), aspartate transferase (AST), lactate dehydrogenase (LDH), cholesterol (CHO), glucose (GLU), inorganic phosphorus (IP), calcium (CA), creatinine (CRE), urea (UREA) and total protein (TPRO) levels. The serum biochemical indicators were assessed with a chemical analyzer, the Architect C8000, series AS1242 (Abbott Diagnostics, Lake Forest, IL, USA).

The fixed environmental factors considered were sex (i.e., male or female), birth type (i.e., singlet or twin), herd (i.e., one of the three herds), feeding type (i.e., feedlot or pasture) and the age of the lamb in days (covariate). The descriptive statistics, data cleaning and linear model fitting were performed in the R statistical environment [37]. Phenotypic distributions of each trait were visually inspected, and outliers with observations deviating three standard deviations ± the mean for each trait were excluded from further analyses. Furthermore, the heteroscedasticity of variances was tested with the Breusch–Pagan test [38]. Additionally, since most of the serum biochemical parameters showed skewed distributions, Box–Cox transformation was applied to the traits of interest preceding model fitting, genetic parameter estimates and association analysis [39]. The descriptive statistics of the phenotypic observations after the outliers were removed are provided in Table 1. Phenotypic correlations among the traits were obtained as pairwise Pearson’s correlation coefficients, and they are given in Table 2.

### 2.2. Genotyping and Quality Control

DNA of the studied animals was extracted from blood samples using a QIACube HT instrument and a Blood/Tissue DNA extraction kit following the manufacturer’s protocol (Qiagen, Hilden, Germany). After obtaining high-quality DNA from each sample, single-nucleotide polymorphism (SNP) genotyping was carried out with the Axiom™ Ovine 50 K SNP Genotyping Array on the GeneTitan™ Multi-Channel Instrument (ThermoFisher Scientific, Waltham, MA, USA), following the manufacturer’s guidelines (Axiom™ 2.0 Assay 96-Array Format Manual Workflow; ThermoFisher Scientific, Waltham, MA, USA). A quality control (QC) followed genotyping, where SNPs that had minor allele frequencies and call rates below 0.05 and 0.95, respectively; deviated from the Hardy–Weinberg Equilibrium (0.05/SNP numbers); and were mapped to sex chromosomes were excluded from further analysis. Additionally, animals with excessively high heterozygosity (false discovery rate (FDR) < 1%), a call rate below 0.90 and an identity by state (IBS) above 0.95 were set to be omitted, though no animals were lost at this stage. A total of 40,868 SNPs passed the QC criteria. All QC processes were undertaken with the ‘GenABEL’ R package version 3.6.3 [40].

### 2.3. Estimation of Genetic Parameters

Univariate and bivariate analyses of linear animal mixed models were implemented to obtain genomic heritability estimates and pairwise genetic correlations, respectively, for the serum biochemical traits, which are presented in Table 3. The model description and variance–covariance structure of the estimations, which were carried out using the ‘sommer’ R package version 4.3.4, are detailed below [41]:

y=Xβ+Zu+e

$$V=\left[\begin{array}{ccc} Z_{i}G\sigma_{u_{i}}^{2}Z_{i}^{\prime }+I\sigma_{e_{i}}^{2} & \cdots & Z_{i}G\sigma_{u_{i}}^{2}Z_{i}^{\prime }+I\sigma_{e_{i}}^{2}\\ \vdots & \ddots & \vdots \\ Z_{i}G\sigma_{u_{i,j}}Z_{j}^{\prime }+I\sigma_{e_{i,j}} & \cdots & Z_{j}G\sigma_{u_{i,j}}^{2}Z_{j}^{\prime }+I\sigma_{e_{j}}^{2} \end{array}\right]$$

where y is the vector of observations; β is the vector of significant fixed effects to be accounted for in the model (i.e., significant environmental factors after model fitting); u and e are the random effects of additive genomic breeding values and residual errors that are assumed to be drawn from an MVN (0, $G\sigma_{u}^{2}$) and an MVN (0, $I\sigma_{e}^{2}$), respectively; X and Z are the matrices mapping the fixed effects and the breeding values to the observations of the traits considered; $\sigma_{u}^{2}$ and $\sigma_{e}^{2}$ are the additive genetic variance and environmental variance for each trait of interest; the ‘i’ and ‘j’ superscripts are the specific traits recorded for each animal that are handled for bivariate analyses; and, finally, I is an identity matrix while G is the genomic relationship matrix (GRM) obtained by ‘Model 1’ described in [42]. Fixed environmental effects were not included in the bivariate analysis to avoid convergence problems. The (co-)variance components of the models for univariate and bivariate analyses were estimated with the Newton–Raphson optimization approach to direct inversion (DI)-based restricted maximum likelihood (REML) using the GRM provided [43,44]. Standard errors of genetic correlations were estimated with the delta method by a second-order Taylor series expansion [45]. Table 2 provides the estimated heritabilities (on the diagonal), genetic correlations (below the diagonal) and phenotypic correlations (above the diagonal).

### 2.4. Genome-Wide Association Studies (GWASs)

In the current study, ten serum biochemical indicators (see Table 1) of Akkaraman lambs were selected as phenotypes for GWAS analysis. Following the genetic parameter estimation, univariate genome-wide association studies were implemented for each trait by using the linear mixed model and the GRM described above to avoid bias and minimize false-positive rates due to population stratification and cryptic relatedness [46,47]. The same significant environmental factors were used, and the SNPs were fitted as fixed factors consecutively, one at a time, each assuming a trend for the copy number of the minor allele (i.e., ‘0’ for homozygous major alleles and ‘1’ for heterozygous and ‘2’ for homozygous minor alleles) for holding additivity. Details of the mixed model-based association test and its previous applications can be found in [24,47]. Concisely, it is an animal mixed model approach to family-based association tests that accommodates a GRM weighted with allele frequencies to avoid inflation of the test statistics because of possible population stratification and relatedness, as originally proposed by [47]. Following the association tests, the inflation factor (λ) and quantile–quantile plots of the *p*-values were obtained. ‘Genomic control’ correction was utilized to further adjust for any possible inflation of the test statistics by setting λ to 1 [48]. The *p*-values of the SNPs were illustrated as ‘−log10 (*p*-values)’ on Manhattan plots for each trait, considering the corresponding chromosome, while two significance thresholds were imposed to detect genome-wide significance (i.e., solid lines) and genome-wide suggestive significance (i.e., dashed lines). To prevent false associations due to multiple testing, Bonferroni correction was applied to the significance thresholds. This correction involved dividing the initial significance level of 0.05 by the total number of SNPs that passed the quality control (40,868 SNPs) for genome-wide significance, while this value was multiplied with the number of chromosomes to obtain the representative chromosome-wide significance. Thus, the genome-wide significance threshold was 1.223 × 10^−06^ and the chromosome-wide significance was 3.181 × 10^−05^, the values of which were 5.91 and 4.50, respectively, on the −log10 (*p*-value) scale on the Manhattan plots. All the steps of the genome-wide association analysis for each trait were carried out using the ‘GenABEL’ R package version 3.6.3 [40].

### 2.5. Functional Annotation Analysis

Genomic positions and nearby genes related to associated SNPs were retrieved from the Oar_v4.0 genome assembly on the NCBI Genome Data Viewer [49]. Genes that directly contained significant SNPs were suggested as candidates. However, when the SNP was not within a described gene, the area of the chromosome covering nearly ±500 Kbp from the identified SNP was scanned for the nearest candidate gene with a reasonable explanation. Identified genes were functionally enriched to recover biological information and the KEGG (Kyoto Encyclopedia of Genes and Genomes) pathways involved using the Database for Annotation, Visualization, and Integrated Discovery (DAVID) Bioinformatics Resources 2021 [50,51]. Where the sheep genome suffers from lack of annotation, orthology among species was exploited to annotate relevant genes from other species, such as cattle, mice and humans. The biological processes of the identified genes were given with their Gene Ontology (GO) terms, and further details can be found on QuickGO provided by the EMBL’s European Bioinformatics Institute [52]. Finally, an animal QTL database was scanned to identify whether the SNPs detected in this study were previously associated with any serum biochemical traits [53].

## 3. Results

### 3.1. Phenotypic Correlation between and Genetic Parameter Analyses of Serum Biochemical Indicators

Ten serum biochemical parameters were investigated in this study, including ALT, AST, CHO, LDH, CA, IP, CRE, GLU, TPRO and UREA. The descriptive statistics for the serum biochemical indicators are listed in Table 1. Table 2 shows the genetic correlations with their standard errors (below the diagonal) and phenotypic correlations with standard errors (above the diagonal) between serum biochemical indicators to provide a context for the use of blood serum components in sheep breeding. The values on the diagonal represent narrow-sense genomic heritability ($h^{2}$) estimates of the ten traits. A wide range of Pearson correlation coefficients were observed, ranging from −0.04 to 0.91, among the phenotypic measurements of serum biochemical indicators. The phenotypic correlation results showed that ALT, AST, CHO and CA were strongly positively correlated, as were AST, LDH, TPRO and CRE. In contrast, traits such as IP and GLU were negatively correlated with UREA (Table 2).

Narrow-sense genomic heritability estimates are presented on the diagonal of Table 2. Overall, low to moderate heritability estimates were found, ranging between 0.14 and 0.55 for serum biochemical indicators (Table 2). The trait demonstrating the highest heritability was TPRO (h^2^ = 0.55), while the serum concentration of AST had the lowest heritability (h^2^ = 0.14). Heritabilities for the traits TPRO (0.55), CHO (0.43) and LDH (0.36) can be classified as moderate, while ALT (0.21), AST (0.14), CA (0.27), IP (0.29), CRE (0.20), GLU (0.15) and UREA (0.18) had low estimates. Genetic correlations were estimated using multivariate mixed linear models, and they are shown in the lower diagonals in Table 2. The trait TPRO showed high genetic correlations with LDH, CHO and AST (r = 0.98, 0.81 and 0.81, respectively). The serum concentration of UREA was found to have negative genetic correlations with the levels of GLU and IP (r = −0.63 and −0.52, respectively; Table 2).

### 3.2. Genome-Wide Association Studies (GWASs)

**Figure 1:**
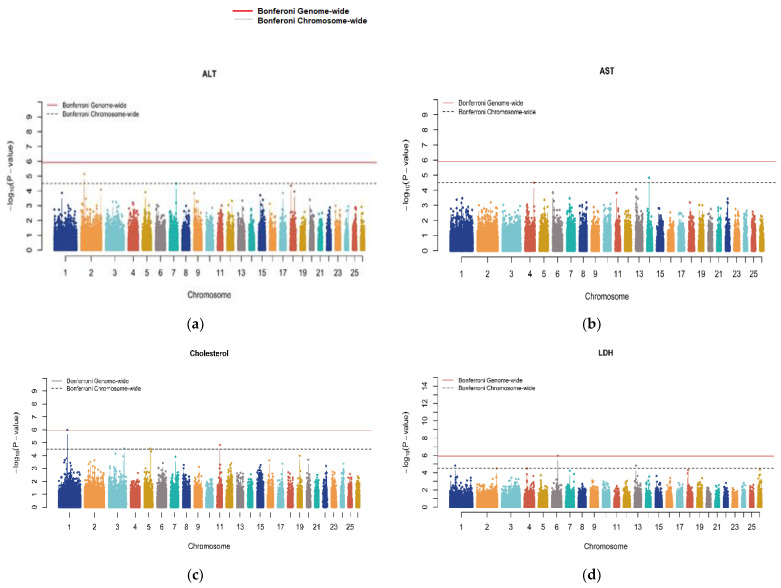

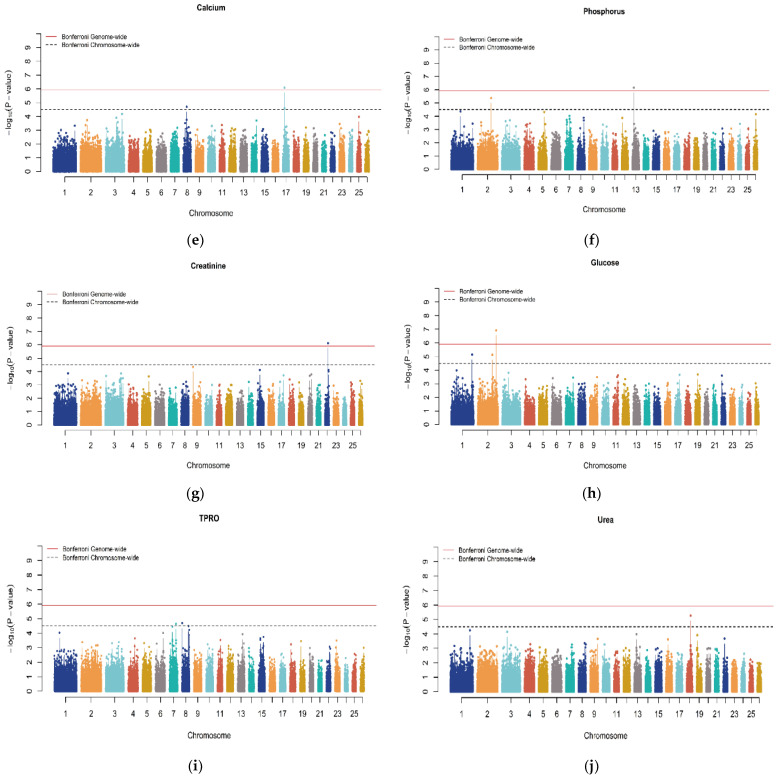
Manhattan plots for all serum biochemical indicators. Genome scaffolds sorted by length were numbered starting at 1. The red lines denote a genome-wide significance threshold (*p* = 1.223 × 10^−06^), and the dashed lines denote a suggestive (chromosome-wide) significance threshold (*p* = 3.181 × 10^−05^).

Genome-wide significant signals in Manhattan plots were only observed for traits encompassing CHO, CA, CRE, GLU, LDH and IP (Figure 1). The Q-Q plots (Supplementary Figure S1) showed that the model we used was reasonable; most of the observed *p*-values were consistent with the expected values, and significant SNPs were found, indicating that the above association analysis results for serum parameter traits are reliable. All traits were forced to have a lambda (λ) of approximately 1 by correcting the *p*-values with the genomic control.

The significant putative QTLs with the candidate genes and the top associated SNPs within each region are shown in Table 3 and Table 4. A total of 23 significant loci were identified, of which 6 were genome-wide (GW) significantly associated with CHO, CA, CRE, GLU, LDH and IP (Table 3) and 17 were chromosome-wide (CW) significantly associated with CHO, ALT, AST, CA, GLU, LDH, IP, TPRO and UREA (Table 4). CHO has four putative QTLs distributed in four ovine chromosomes (OAR). For CHO, the top significant locus (rs415766081, with a *p* = 1.022 × 10^−06^) was in the intron region of the Spectrin α, erythrocytic 1 (*SPTA1*) gene. A GW-significant SNP for CA was identified on OAR17 (rs427096440, with a *p* = 8.033 × 10^−07^) in the vicinity of the microsomal glutathione S-transferase 2 (*MGST2*) gene. The GW-significant SNP for CRE (rs423178582, with a *p* = 7.716 × 10^−07^) was identified on OAR22, which is 42 Kb from the CDK2-associated cullin domain 1 (*CACUL1*) gene. The SNP rs428784360 (*p*-value = 1.207 × 10^−07^) GW significantly associated with GLU is located on OAR2 (Table 3). This marker is located within the intron of the ENSOARG00020040484.1 gene. Another SNP (rs410665381, with a *p* = 1.216 × 10^−06^) was found to be GW-associated with LDH. The locus was observed to be located 267 Kb upstream of insulin-like growth factor binding protein 7 (*IGFBP7*) on OAR6. Finally, another GW-significant SNP (rs404995480, with a *p* = 6.902 × 10^−07^) was associated with IP within an intron of the gene Par-3 family cell polarity regulator (*PARD3*) on OAR13. Additional information on loci with GWS associations is shown in Table 4.

## 4. Discussion

Despite the utmost importance of serum indicators such as lipids, proteins, enzymes, minerals and metabolites in livestock production systems, very few studies have investigated the underlying genetic architecture and mechanisms behind these complex traits [5,7,33,34]. In this investigation, we measured 10 serum biochemical indicators. Certain serum biochemical indicators demonstrated strong phenotypic and genetic correlations among each other. To the best of our knowledge and according to an animal QTL database, no published study has systematically demonstrated the genetic parameters of some or all of the 10 serum biochemical indicators and genomic loci using a GWAS of SNPs in lambs [53]. Additionally, only one study was observed to have focused on the genetic basis of protein levels in sheep [12]. The aim of the current study was to identify the underlying genetic architecture for blood serum indicators in lambs. ALT and AST averages were in the range of previous reports for Akkaraman lambs and other breeds, such as Ba sheep, Karakul and Tzurcana ewes, Balami ewes, Lori-Bakhtiari and Mehraban sheep, and Santa Inês ewes. Contrarily, a low heritability estimate was detected for UREA in Santa Inês sheep [54,55].

Genetic variance in serum parameters plays a crucial role in understanding animals’ ability to combat infections and stress. This insight can aid in devising better strategies to enhance disease resistance and resilience [7]. The identified low to moderate genomic heritability estimates indicate the potential of genomic selection to result in a gradual improvement in breeding programs in sheep. In the present study, heritabilities were estimated for, for instance, TPRO (0.55 ± 0.14), UREA (0.18 ± 0.11), LDH (0.36 ± 0.14) and ALT (0.14 ± 0.10) (Table 2), which indicated considerable genetic effects on these protein fractions and probably their potential use as biomarkers for genetic selection. This result differs from the reported studies on Lori-Bakhtiari sheep, where genomic heritability was found to be low (0.00 ± 0.29), possibly due to the limited number of animals, which caused high standard errors in the heritability estimates [12]. Similarly, a low heritability estimate was detected for UREA in Santa Inês sheep and in Holstein-Friesian cows [55,56]. Our study suggests a genomic heritability estimate for serum CA of 0.27 ± 0.13 in Akkaraman lambs, which is higher than that reported for cattle [56]. The current study is the first to focus on the genetic parameters of a wide range of serum biochemical indicators for Akkaraman sheep and one of the very first on global sheep populations. However, further research is still required to determine the genetic background of blood serum indicators precisely, as indicated by the slightly high standard errors of the heritability estimates, which was due to the relatively low number of animals studied.

Multiple candidate genes were identified in the present study (Table 3 and Table 4). A key result is the enrichment of biological processes for the candidate genes that aid disease response and immune system regulation. Many candidate genes suggested by our study are predicted to be part of biological processes, such as physiological responses to stimuli (GO:0050896), the glutathione biosynthetic process (GO:0006750), the VEGF-activated neuropilin signaling pathway (GO:0038190), regulation of the apoptotic process (GO:0042981), regulation of the metabolic process (GO:0019222), regulation of the immune system process (GO: 0002682), immune response (GO:0006955), regulation of response to stress (GO:0080134), cell communication (GO:0007154) and regulation of signaling (GO:0023051), in various organisms. Additionally, some of the candidate genes were predicted to have molecular functions, such as catalytic and transferase activities, as well as ion, small molecule and enzyme binding.

A genome-wide associated SNP (rs415766081, *p* = 1.022 × 10^−06^; Table 3) was located in the intron of the Spectrin α, erythrocytic 1 (*SPTA1*) gene on OAR1 for CHO. Cholesterol is a vital molecule for membrane fluidity, permeability, gene transcription, growth and development, and it serves as the backbone of steroid hormones and vitamin D analogs [57]. Functional annotation of *SPTA1* shows that it is involved in actin cytoskeleton organization (GO:0030036), the immune system process (GO:0002376), lymphocyte (GO:0002260) and leukocyte homeostasis (GO:0001776), and positive regulation of T-cell proliferation (GO:0042102) and that it has molecular functions, such as actin filament binding (GO:0051015) and calcium-ion binding (GO:0005509), in various mammals. KEGG enrichment also shows that it is involved in apoptosis. Both actin filament organization and calcium have long been recognized for their critical role in serum cholesterol levels [58,59]. Orthologs of this gene have been associated with increased B cell numbers, IgG levels and T cell numbers in mice [60].

Another genome-wide associated SNP on OAR17 was found 31 Kb from the microsomal glutathione S-transferase 2 (*MGST2*) gene for CA (Table 2). *MGST2* is a member of the superfamily MAPEG (membrane-associated proteins in eicosanoid and glutathione metabolism), and it has a role in the interactions between proteins that detoxify highly reactive lipophilic substances and proteins involved in the endogenous metabolism of reactive lipophilic intermediates (leukotrienes) [61]. Functional enrichment showed that *MGST2* is involved in eicosanoid metabolic processes (GO:0006690), specifically the leukotriene metabolic process (GO:0006691) and the glutathione biosynthetic process (GO:0006750), as well as response to stress (GO:0006950), defense response (GO:0006952) and inflammatory response (GO:0006954) in various mammals, including sheep. *MGST2* has been annotated by KEGG as being involved in glutathione metabolism, drug metabolism and resistance, chemical carcinogenesis by receptor activation, as well as fluid shear stress and atherosclerosis. *MGST2* is a mainly glutathione-dependent peroxidase and cytoprotective glutathione S-transferase, and it is highly homologous with Leukotriene C4 Synthase (*LTC4S*) [62]. Eicosanoid metabolism, in terms of functional coupling of calcium-dependent phospholipase A2 (*cPLA2*), plays a role in the regulation of intracellular Ca2+ concentration [63]. It is worth noting that an association between *LTC4S* promoter polymorphism and coronary artery calcium thickness was identified in women [62].

The genome-wide associated SNP for serum creatinine levels is located 42 Kb upstream of *CACUL1* (CDK2 associated cullin domain 1) on OAR22. *CACUL1* is predicted to engage in a wide range of organic-substance metabolic processes (GO: 0071704), such as proteolysis (GO: 0006508), positive regulation of cell population proliferation (GO:0008284) and protein kinase activity (GO: 0045860), with its ubiquitin protein ligase- and protein kinase-binding activities. Serum creatinine, as a waste product of muscle metabolism, is one of the primary indicators of renal dysfunction and impaired filtration [64]. Various CDKs (cyclin-dependent kinases) have previously been associated with kidney functions, including cell proliferation and filtration in humans [65,66]. Additionally, a study in mice showed that increased expression of *CDK2* protects podocytes (i.e., a layer of cells around the glomerulus in which filtration of blood takes place) from apoptosis, while reduced expression of *CDK2* leads to increased susceptibility to diabetic nephropathy [67].

The genome-wide associated SNP for serum glucose (rs428784360) was at an intron of ENSOARG00020040484.1 (Table 3). This long noncoding RNA has few annotated functions as yet. The only other gene nearby is LOC121818761, for which there is RNA evidence but little by way of functional assignment as yet. To our knowledge, this study is the first report to link these genes to blood glucose. Further work will be required to investigate their connection to blood glucose and diabetes.

Insulin-like growth factor binding protein 7 (*IGFBP7*) on OAR6 was associated with serum lactate dehydrogenase (LDH) levels. LDH participates in carbohydrate metabolism by facilitating the conversions of lactate and pyruvate using the NAD+/NADH coenzyme system. *IGFBP7* takes part in diverse biological processes, such as the regulation of cell growth (GO:0001558), response to stimuli (GO:0048583), signaling (0023051), as well as the regulation of the steroid metabolic process (GO:0019218) and response to corticosteroids (GO:0031969), glucocorticoids (GO:0051384), chemicals (GO:0042221) and steroid hormones (GO:0048545). Its protein shows molecular functions, such as insulin-like growth factor binding and structural molecule activity. IGFBPs and IGFs have been consistently documented to play a pivotal role in the immune responses of animals and humans [68,69,70]. Elevated plasma *IGFBP7* levels were recently found to be correlated with chronic inflammation in humans [71]. Additionally, IGFs are known to have a regulatory role in glucose uptake and glycogen and lactate metabolism, especially in the Warburg effect (i.e., preferential breakdown of glucose into lactate, even when mitochondria are operating normally), where LDH is also a key enzyme [72,73]. Therefore, *IGFBP7*, as a regulator of IGFs, can be suggested to have an indirect role in regulating serum LDH levels.

Finally, a putative QTN (rs404995480, *p* = 6.902 × 10^−07^; Table 3) was detected in the intron of the Par-3 Family Cell Polarity Regulator (*PARD3*) gene on OAR13 for IP. *PARD3* is known to have significant roles in cytoskeleton organization (GO:0007010), the establishment of cell polarity (GO:0030010), organelle organization (GO:0006996) and the establishment of localization in cells (GO:0051649) with its molecular functions, such as protein binding and phosphatidylinositol binding. It is annotated by KEGG to have roles in Rap1 and chemokine signaling pathways, endocytosis and the Hippo signaling pathway, as well as cell junctions. Inorganic phosphorus plays numerous metabolic roles as a reactant (e.g., glycolysis and oxidative phosphorylation) and product (e.g., nucleic acid synthesis and ATPases) and is recognized as a signaling molecule as well [74]. Many studies have suggested that *PARD3* is regulated by phosphorylation [75]. *PARD3* regulates the initial cell polarity cues. Cell polarity, the asymmetric distribution of proteins, organelles and the cytoskeleton, plays an important role in development, homeostasis and disease [76]. *PARD3*, as a member of the PAR complex, can regulate vesicle transport and control the localization of cytoplasmic proteins, primarily by regulating phosphoinositides [77]. Phosphoinositides, in which one of the isomers is a phosphate, serve as docking sites for proteins in the cell membrane, and their state of phosphorylation determines which proteins can bind to them [78].

Taken together, our results suggest 6 genome-wide and 17 chromosome-wide SNPs and 19 candidate genes, as well as 4 uncharacterized regions, underlying 10 serum biochemical parameters. The relevance and importance of the suggested candidate genes to the immune system, defense response, cytoskeleton organization and other biological processes are mostly characterized in various species. Simultaneously, our study revealed various genetic parameters and phenotypic correlations for these serum biochemical indicators in sheep. None of the associated SNPs have previously been linked to serum biochemical traits in sheep, mainly because there has been only one GWAS implemented only for protein levels in sheep. Therefore, the results of this study can be used to shed light on the research on the molecular mechanisms underlying serum biochemical traits in sheep, which are directly related to the welfare and health status of animals and are indirectly of high economic importance for sheep production systems. Moreover, as sheep can serve as a model organism for studying welfare and diseases relevant to humans, our results may also apply to medical research in human health. In any case, further molecular and population-based validation studies are required to prove the causality of the associated SNPs and suggested genes for their use in sheep genetic improvement programs, gene editing studies and targeted drug applications that aim to improve the immune system, health and welfare in humans and sheep.

## 5. Conclusions

In this work, we report for the first time GWASs together with genetic parameter estimations for levels of serum biochemical indicators in sheep. Detection of QTLs for serum biochemical parameters, due to their strong relationship with many disorders, has unique potential for disease intervention and targeted drug applications. Our research offers genetic tools for additional investigations into causal linkages for particular cases; nonetheless, mechanistic and experimental investigations are necessary to identify the underlying causal chains behind these intricate associations. In total, 23 SNP loci were associated with serum biochemical indicators, leading to 19 candidate genes as well as 4 uncharacterized regions suggested to underly 10 serum biochemical parameters. These are *SPTA1*, *MGST2*, *CACUL1*, *IGFBP7*, *PARD3*, *PHB1*, *SLC15A5*, *TRIM35*, *RGS6*, *NUP93*, *CNTNAP2*, *SLC7A11*, *B3GALT5*, *DPP10*, *HST2ST1*, *NRP1*, *LRP1B*, *MAP3K9* and *ENSOARG00020040484.1*, as well as *LOC101103187*, *LOC101117162*, *LOC105611309* and *LOC101118029.* The current findings provide a comprehensive inventory of the relationships between serum components as well as genetic variants for disease-relevant characteristics. This information may facilitate the identification of therapeutic targets and fluid biomarkers and establish a strong framework for comprehending the pathobiology of complex diseases while highlighting specific loci for targeted genome editing or gene knockout studies. However, more research is needed to identify the specific functional mutations in linkage disequilibrium with the markers in this study. In addition, the functional mutations will need to be validated and examined for potential correlated responses to selection, including production and reproduction traits as well as disease and parasite resistance in sheep.

## Figures and Tables

**Table 1:** Descriptive statistics of serum biochemical indicators in Akkaraman sheep.

Traits	N	Minimum	Mean	SD	Maximum
ALT	417	5	12.64	4.21	27
AST	393	40	78.04	18.24	141
CHO	415	21	45.99	14.35	85
LDH	400	228	494.69	111.48	912
CA	415	5.20	8.11	1.02	13.10
IP	410	2.90	5.85	1.04	9.60
CRE	422	0.33	0.53	0.08	0.91
GLU	422	29	60.11	10.51	92
TPRO	418	36.29	54.11	7.39	76.96
UREA	414	11	43.01	9.39	84

Notes: ALT = alanine aminotransferase (U/L); AST = aspartate aminotransferase (U/L); CHO = cholesterol (mg/dL); LDH = lactate dehydrogenase (U/L); CA = calcium (mg/dL); IP = inorganic phosphorus (mg/dL); CRE = creatinine (mg/dL); GLU = glucose (mg(dL); TPRO = total protein (g/L); UREA = urea (mg/dL). Additionally, SD refers to the standard deviation and N stands for sample size.

**Table 2:** Heritability and genetic and phenotypic correlations of serum biochemical indicators in Akkaraman sheep. Diagonal values (bold) represent the genomic heritability of the overlapping trait, where phenotypic correlations (±standard errors) are given above the diagonal and genetic correlations (±standard errors) are given below the diagonal.

Traits	ALT	AST	CHO	LDH	CA	IP	CRE	GLU	TPRO	UREA
ALT	**0.21 ± 0.11**	0.48 ± 0.04	0.18 ± 0.04	0.49 ± 0.04	−0.01 ± 0.05	0.22 ± 0.04	−0.01 ± 0.05	0.02 ± 0.05	0.35 ± 0.04	0.09 ± 0.05
AST	0.91 ± 0.08	**0.14 ± 0.10**	0.40 ± 0.04	0.53 ± 0.04	0.28 ± 0.04	0.18 ± 0.04	0.30 ± 0.04	0.14 ± 0.05	0.47 ± 0.04	0.27 ± 0.04
CHO	0.48 ± 0.16	0.77 ± 0.12	**0.43 ± 0.14**	0.35 ± 0.04	0.19 ± 0.04	0.26 ± 0.04	0.20 ± 0.04	0.19 ± 0.04	0.54 ± 0.04	0.32 ± 0.04
LDH	0.82 ± 0.08	0.87 ± 0.07	0.70 ± 0.13	**0.36 ± 0.14**	0.14 ± 0.05	0.32 ± 0.04	0.14 ± 0.04	0.20 ± 0.04	0.52 ± 0.04	0.07 ± 0.06
CA	−0.03 ± 0.20	0.64 ± 0.12	0.39 ± 0.11	0.36 ± 0.14	**0.27 ± 0.13**	0.11 ± 0.05	0.99 ± 0.01	0.24 ± 0.04	0.35 ± 0.04	0.28 ± 0.04
IP	0.84 ± 0.23	0.88 ± 0.33	0.53 ± 0.14	0.80 ± 0.15	0.37 ± 0.17	**0.29 ± 0.13**	0.10 ± 0.05	0.19 ± 0.04	0.36 ± 0.04	−0.10 ± 0.05
CRE	−0.04 ± 0.22	0.66 ± 0.12	0.42 ± 0.11	0.28 ± 0.14	0.11 ± 0.06	0.35 ± 0.19	**0.20 ± 0.11**	0.23 ± 0.04	0.34 ± 0.04	0.29 ± 0.04
GLU	0.13 ± 0.23	0.77 ± 0.34	0.54 ± 0.19	0.78 ± 0.25	0.61 ± 0.14	0.76 ± 0.24	0.62 ± 0.15	**0.15 ± 0.10**	0.23 ± 0.04	−0.11 ± 0.05
TPRO	0.71 ± 0.12	0.81 ± 0.09	0.81 ± 0.07	0.98 ± 0.08	0.64 ± 0.09	0.80 ± 0.13	0.64 ± 0.10	0.59 ± 0.17	**0.55 ± 0.14**	0.21 ± 0.04
UREA	0.27 ± 0.32	0.73 ± 0.17	0.74 ± 0.14	0.29 ± 0.34	0.76 ± 0.16	−0.52 ± 0.38	0.78 ± 0.15	−0.63 ± 0.46	0.59 ± 0.18	**0.18 ± 0.11**

ALT = alanine aminotransferase (U/L); AST = aspartate aminotransferase (U/L); CHO = cholesterol (mg/dL); LDH = lactate dehydrogenase (U/L); CA = calcium (mg/dL); IP = inorganic phosphorus (mg/dL); CRE = creatinine (mg/dL); GLU = glucose (mg(dL); TPRO = total protein (g/L); UREA = urea (mg/dL).

**Table 3:** Genome-wide significant SNP markers for serum biochemical indicators.

Trait	SNP	Chr	Oar_v4.0 Position (bp)	*p*-Value	MAF	Effect Size	Candidate Gene	Distance
CHO	*rs415766081*	1	107,828,780	1.022 × 10^−06^	0.110	0.084	Spectrin α, erythrocytic 1 (*SPTA1*)	Intron variant
CA	*rs427096440*	17	17,753,256	8.033 × 10^−07^	0.414	0.004	Microsomal glutathione S-transferase 2 (*MGST2*)	~31 Kb upstream
CRE	*rs423178582*	22	37,960,974	7.716 × 10^−07^	0.157	0.068	CDK2 associated cullin domain 1 (*CACUL1*)	~42 Kb upstream
GLU	*rs428784360*	2	227,357,948	1.207 × 10^−07^	0.160	0.092	*ENSOARG00020040484.1*	Intron variant
LDH	*rs410665381*	6	72,632,996	1.216 × 10^−06^	0.129	0.117	Insulin-like growth factor binding protein 7 (*IGFBP7*)	~267 Kb upstream
IP	*rs404995480*	13	17,678,848	6.902 × 10^−07^	0.388	0.063	Par-3 family cell polarity regulator (*PARD3*)	Intron variant

Chr = chromosome; MAF = minor allele frequency; CHO = cholesterol; CA = calcium; CRE = creatinine; GLU = glucose; LDH = lactate dehydrogenase; IP = phosphorus.

**Table 4:** Chromosome-wide significant SNP markers for serum biochemical indicators.

Trait	SNP	Chr	Oar_v4.0 Position (bp)	*p*-Value	MAF	Effect Size	Candidate Gene	Distance
CHO	rs415259159	11	36,648,365	1.536 × 10^−05^	0.417	0.047	Prohibitin 1 (*PHB1*)	~35 Kb upstream
CHO	rs408900631	3	198,343,644	2.820 × 10^−05^	0.432	0.047	Solute Carrier Family 15 Member 5 (*SLC15A5*)	~55 Kb downstream
CHO	rs403535835	5	75,927,368	2.923 × 10^−05^	0.101	0.073	*LOC101117162*	~19 Kb upstream
ALT	rs413251030	2	38,421,272	7.175 × 10^−06^	0.194	0.380	Tripartite Motif Containing 35 (*TRIM35*)	~71 Kb downstream
ALT	rs421887664	7	80,842,728	3.158 × 10^−05^	0.158	0.439	Regulator of G-Protein Signaling 6 (*RGS6*)	Intron variant
AST	rs405842437	14	24,175,813	1.453 × 10^−05^	0.449	0.013	Nucleoporin 93 (*NUP93*)	Intron variant
AST	rs423986212	4	109,758,783	3.111 × 10^−05^	0.269	0.014	Contactin Associated Protein 2 (*CNTNAP2*)	Intron variant
CA	rs421266853	8	39,031,937	1.967 × 10^−05^	0.417	0.003	*LOC105611309*	~56 Kb downstream
CA	rs408365736	17	19,135,137	2.576 × 10^−05^	0.077	0.006	Solute Carrier Family 7 Member 11 (*SLC7A11*)	Intron variant
GLU	rs412782784	1	257,987,356	7.143 × 10^−06^	0.067	0.118	β-1,3-Galactosyltransferase 5 (*B3GALT5*)	~288 Kb downstream
GLU	rs410943504	2	178,724,382	7.415 × 10^−06^	0.457	0.059	Dipeptidyl Peptidase Like 10 (*DPP10*)	Intron variant
LDH	rs402703943	1	63,683,463	1.665 × 10^−05^	0.218	0.090	Heparan Sulfate 2-O-Sulfotransferase 1 (*HST2ST1*)	~185 Kb downstream
LDH	rs410138359	13	18,806,069	1.678 × 10^−05^	0.169	0.087	Neuropilin 1 (*NRP1*)	~44 Kb downstream
IP	rs420848991	2	168,420,121	4.201 × 10^−06^	0.191	0.077	LDL Receptor Related Protein 1B (*LRP1B*)	Intron variant
TPRO	rs423075621	8	963,780	2.017 × 10^−05^	0.488	0.045	*LOC101118029*	~74 Kb upstream
TPRO	rs401111582	7	79,289,788	2.319 × 10^−05^	0.386	0.044	Mitogen-Activated Protein 3 Kinase 9 (*MAP3K9*)	Intron variant
UREA	rs403791299	18	48,405,490	5.277 × 10^−06^	0.432	1.971	*LOC101103187*	~127 Kb upstream

Chr = chromosome; MAF = minor allele frequency; CHO = cholesterol; ALT = alanine aminotransferase; AST = aspartate aminotransferase; CA = calcium;; GLU = glucose; LDH = lactate dehydrogenase; IP = phosphorus; TPRO = total protein; UREA = urea.

## Data Availability

The original contributions presented in the study are included in the article/Supplementary Material, further inquiries can be directed to the corresponding author.

## Supplementary Materials

The following supporting information can be downloaded at: https://www.mdpi.com/article/10.3390/genes15080990/s1, Figure S1: Quantile–quantile (Q–Q) diagram of the effects of population stratification.
