# Supplementary material for: Unravelling the Genetic Architecture of Serum Biochemical Indicators in Sheep
Source: Genes (Basel). 2024 Jul 27;15(8):990. doi: 10.3390/genes15080990 (PMC11353979; doi:10.3390/genes15080990)
Supplement: Supplementary file 1 [file genes-15-00990-s001.zip › genes-3090923-Supplementary.pdf]

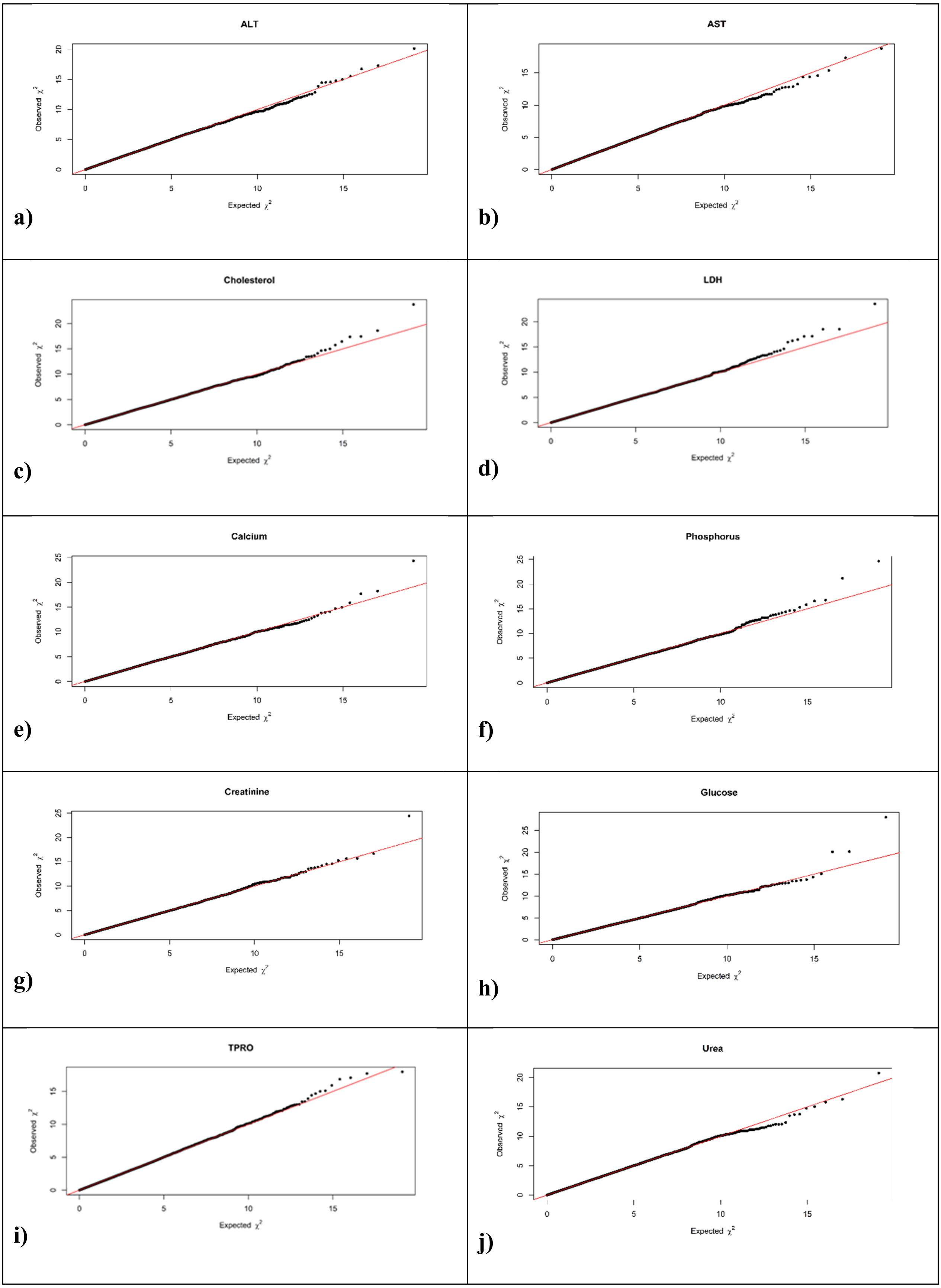
ALT
AST
Cholesterol
LDH
Calcium
Phosphorus
Creatinine
Glucose
TPRO
Urea
Observed $\chi^2$
Expected $\chi^2$
a)
b)
c)
d)
e)
f)
g)
h)
i)
j)

**Supplementary Figure S1** Quantile–quantile (Q–Q) diagram of the effects of population stratification. Q–Q plot showing the late separation between observed and expected values. The red lines indicate the null hypothesis of no true association. Deviation from the expected *p*-value (expressed as $-\log_{10}(p)$ distribution is evident only in the tail area for serum biochemical indicators, indicating that population stratification was properly controlled.
